# Economic Development, Fiscal Ecological Compensation, and Ecological Environment Quality

**DOI:** 10.3390/ijerph19084725

**Published:** 2022-04-13

**Authors:** Hongjie Cao, Meina Li, Fengqin Qin, Yankun Xu, Li Zhang, Zhifeng Zhang

**Affiliations:** 1 School of Economics, Qingdao University, Qingdao 266071, China; jaytsoo@qdu.edu.cn (H.C.); 2021020068@qdu.edu.cn (M.L.); 202130274@mail.sdu.edu.cn (L.Z.); 2 Chinese Academy of Fiscal Sciences, Beijing 100142, China; 3 School of Economics, South-Central Minzu University, Wuhan 430074, China; 2019035@mail.scuec.edu.cn

**Keywords:** economic development, fiscal ecological compensation, ecological environment, N-shaped nonlinear relationship

## Abstract

Focusing on the exploration of the important role of fiscal ecological compensation in green development, this paper incorporates fiscal ecological compensation into the analytical framework of green development. Based on samples of data from county areas in China in 2017 and 2018, this paper empirically examines the shape of the green development routes in county areas in China. On this basis, this paper explores the impact and mechanism of fiscal ecological compensation on the green development path in China. The empirical results show that there is a nonlinear, N-shaped relationship between economic development and the ecological environment in China within the range of the sample examined. Fiscal ecological compensation has a direct governance effect on the ecological environment of deterring ecological damage and providing financial compensation. Fiscal ecological compensation has an indirect impact on the ecological management of different regions by influencing economic development. Therefore, while focusing on transforming the economic development model, local governments should adopt policy instruments such as expanding the coverage of financial ecological compensation, deepening the design of the financial ecological compensation system, and systematically evaluating the effects of financial ecological compensation policies. The government should further improve and optimize the fiscal eco-compensation system in order to help China’s green and high-quality development.

## 1. Introduction

Since the reform and opening in 1978, Chinese economy has experienced rapid growth, and its development targets have been achieved. Past practice shows that excessive consumption of resources and environmental degradation are the main problems facing countries at the stage of rapid economic development. Many studies have revealed that there may be a non-linear relationship between economic growth and the ecological environment [1,2,3,4,5]. Among them, the EKC (Environmental Kuznets curve: Panayotou first called this relationship between environmental quality and per capita income the Environmental Kuznets Curve (EKC) in 1993, based on the Kuznets curve proposed by American economist Simon Smith Kuznets in 1955. When a country has a low level of economic development, the level of environmental pollution is less. However, as per capita income increases, environmental pollution tends to increase from low to high, and environmental degradation increases with economic growth. When economic development reaches a certain level, that is, after reaching a certain critical point or “inflection point”, with the further increase in per capita income, environmental pollution tends to decrease from high to low, and the degree of environmental pollution gradually slows down, and the quality of the environment gradually improves. On this basis, this paper further incorporates ecology-related indicators into the core explanatory variables to give a more comprehensive response to the whole picture of ecological environment. In order to reflect the difference caused by this expansion, we define it as the ecological pants Nietzsche curve. However, this definition needs to be further studied and deepened.) theory illustrates that environmental pollution initially shows an upward and downward trend with economic development. Currently, rapid “broad” economic growth has exacerbated resource consumption and the deterioration of the ecological environment, limiting the sustainable development of the Chinese economy and society. Consequently, it is urgent to look for a method of green development.

The three-dimensional co-management framework, “government-corporate-individual”, is considered an ideal model to achieve green development. However, due to the externals of the ecological environment and the limitations of the stage of social development, society and individuals have failed to play an effective role in China’s existing ecological and environmental governance system. The government serves as the most important role in the current eco-environmental governance system. The government directly addresses the ecological environment through energy conservation and environmental protection expenditure and environmental protection taxes on both the fiscal and revenue sides. At the same time, the government also regulates ecological harm administratively through a series of legal systems. In addition, government policies also indirectly impact environmental management by influencing economic development.

Among the many ecological and environmental management measures taken by the government, financial ecological compensation, which regulates the main body of ecological and environmental relations by economic means, has been favored by the government. Fiscal ecological compensation focuses on the ecosystem itself with corresponding fiscal instruments, such as transfers and subsidies. It compensates ecological providers by paying for the additional protection, associated construction costs, and the opportunity costs of development foregone for this purpose. In addition, this policy tool internalizes the externality involved from the standpoint of the royalties of the beneficiaries in order to promote green development. The tax-sharing system based on Ecological Value-Added Tax and Service Tax, first established by the Parana State in Brazil in 1992, can be regarded as the specific application of fiscal ecological compensation in practice. Similarly, the transfer payment system for national key ecological functional areas that China began to pilot in 2008 is also the most important attempt of vertical fiscal ecological compensation in China’s reality. By 2022, more than 800 counties in China will have received ecological transfer payments, with the total size of the transfer payments exceeding CNY 600 billion. According to the corresponding county ecological and environmental governance assessments, national transfer payments to key ecological function areas have had a significant impact on China’s ecological and environmental governance and economic and social development [6].

This paper is based on the background of excessive consumption of resources and a certain degree of deterioration of ecological environment that China has faced along with the rapid economic growth since the reform and opening up. This paper combines the possible non-linear relationship between economic growth and ecological environment found in numerous studies. Under the requirement of green development, how to realize the coordinated and sustainable development of economic growth and ecological environment in the new normal remains an important research theme with distinctive contemporary significance. Accordingly, this paper focuses on the exploration of the important role of fiscal ecological compensation in green development. This paper seeks to analyze the direct and indirect impacts of fiscal ecological compensation systems on ecological governance based on the recognition of the relationship between economic development and the ecological environment.

Firstly, this article complements and tests the fundamental theory of green development. Fiscal ecological compensation is an innovative governance tool that combines market and government means. It is not limited to previous research on the incentive effect and mechanism of fiscal ecological compensation. This paper integrates economic development and the environmental environment under the same analytical framework [5,6]. On this basis, this paper incorporates fiscal eco-compensation factors into the analytical framework to explore the direct and indirect impacts of fiscal eco-compensation on China’s green development. This paper extends somewhat on the theoretical basis, impact, and mechanism of action of theories related to ecological fiscal compensation to promote the path of achievement of green development.

Second, based on a summary of the evolution of China’s green development and fiscal eco-compensation system, this paper examines the relationship between economic and social development and eco-environmental governance and the green development effects of fiscal eco-compensation. The direct and indirect impacts of fiscal eco-compensation on green development are analyzed. The institutional barriers involved are analyzed in order to provide a benchmark for the construction of an eco-compensation system with institutional regulation, efficient incentives, and coordinated development and indirect effects; analyze the existing system and mechanism obstacles; and provide reference for the construction of a fiscal ecological compensation system with institutional norms, incentives, and coordinated development. With appropriate extensions, the research in this paper has important empirical implications for understanding green development in developing countries.

Third, rather than limiting research to parts of China or above the county level, this paper focuses on the county level, which is the foundation of China’s overall economic and social governance. This paper seeks to specifically and precisely measure the relationship between economic development and the ecological environment and to explore the impact of fiscal ecological compensation on China’s green development pathway.

The rest of this paper is arranged as follows: The Section 2 is literature review; Section 3 is empirical design; Section 4 is empirical analysis; and Section 5 is research conclusions and policy suggestions.

## 2. Literature Review

### 2.1. Economic Development and Ecological Environment

The study of the relationship between economic development and environmental sustainability began with Grossman and Krueger. Grossman and Krueger discovered that there is an inverse U-shaped relationship between SO_2_ and soot emissions and economic growth [1]. Panayotou performed a detailed analysis of the inverse U-relationship between economic development and environmental pollution. Then, Panayotou suggested naming the inverted U-curve between the two as “Kuznets Environmental Curve” [2]. Subsequently, studies using different types of pollution discharge indicators, such as air pollution, water pollution, and solid waste pollution, as proxy variables of environmental pollution confirmed the nonlinear relationship between the two [3,4]. Grossman and Krueger presented the mechanism for the impact of economic development on the quality of the environment in terms of scale, technology, and structure [5].

Rapid economic development has led to excessive consumption of natural resources, and predatory exploitation of resources has put severe strain on the ecological environment [7,8]. Based on the research of Grossman and Krueger [1], the (inverted) U-shaped relationship between economic development and resources and ecology is supported by some studies, that is, in line with the traditional EKC hypothesis [9,10,11]. For instance, Madhusudan and Michael used the deforestation rate as a measure of the ecological environment, confirming that there is a strong EKC relationship between the income rate and the deforestation rate [9]. Based on the ecological footprint data of 22 European countries, Saqib and Benhmad empirically tested the quadratic relationship between income growth and ecological footprint [11]; that is, the hypothesis of ecological EKC is supported. At the same time, a large body of literature has found that the relationship between economic development and the ecological environment is not a simple quadratic one. Economic development and ecological environment show a cubic (inverse) N-shaped correlation [12,13,14,15,16]. For example, research results from Zhou et al. show a non-linear, N-shaped relationship between economic development and environmental pollution [13,14]. The empirical results of Kang et al. demonstrate a non-linear, inverted N-shaped relationship between economic development and carbon dioxide emissions [15]. Moreover, some studies do not think that there is a non-linear relationship between the two [17]. Different empirical results may be linked to differences in sample time intervals, substitution variables, and selection of econometric models [18].

### 2.2. Influencing Factors of Nonlinear Relationship Changes

Economic and social factors are major shocks to the relationship between economic development and the ecological environment. Firstly, energy consumption patterns can alter the relationship between economic development and the ecological environment. With the development of the economy, the level of energy consumption of the population will change, initially increasing and then decreasing. When the level of economic development improves, people become more interested in the current and future environmental conditions. In addition, people are more willing to sacrifice energy consumption in return for a high-quality environmental environment. It shifts the inflection point of the non-linear curve between economic development and the ecological environment earlier and mitigates the negative impact of economic development on the ecological environment [19,20]. Secondly, financial development policies such as credit expansion have not only increased consumer demand for energy-intensive products but have also led to more environmental degradation. This in turn shifts the inverted U-shaped curve between economic development and the ecological environment [21,22]. Finally, the economic structure also affects the non-linear relationship between economic development and environmental protection. The modernization of the economic structure will change the “extensive” pattern of development of high pollution, high energy consumption, and high emissions at an early stage of economic development. This will reduce the pressure on the ecosystem caused by economic development and shift the EKC curve to the left [23].

The government’s environment policy is a factor that cannot be ignored to change the curve. Panayotou stressed that improving environmental quality depends primarily on government policies, social systems, and market integrity and functioning rules [24]. When the government has enough information, it can set higher ecological standards and stricter environmental laws and regulations. At the same time, the market uses more technologically advanced technology to produce its products. The second inflection point of the N-shaped curve means that the quality of the ecological environment will improve. The objective of regulating the relationship between economic development and the ecological environment could be achieved through commercial means. For example, carbon trading could reconcile supply and demand between those with a surplus of carbon credits and those with a shortage of carbon credits through market forces. This reduces the distance between the inflection points of the original N-type EKC curve and contributes significantly to the reduction of CO_2_ emissions. A series of pollution-control policies developed by the government will also affect the relationship between economic development and the ecological environment [23]. In general, the use of coercive administrative regulation by the government can also shift the relationship between economic development and the ecological environment in a direction that is conducive to improving the ecological environment. For example, environmental regulations may have a significant impact on the peak and position of the EKC, which causes the EKC to move down to the left. However, the direction of government regulation of the relationship between economic development and the ecological environment through economic instruments such as green fiscal revenues and expenditures is uncertain. There are both positive and negative impacts. For example, green fiscal revenues and expenditures have a negative effect on carbon emissions. The cumulative effect of green finance on environmental protection has yet to be published, with China only recently involved in the economic sphere. In addition, there is a problem of moral hazard due to information asymmetry between enterprises and government. This has led some enterprises to increase their pollution emissions after obtaining green fiscal support. This ultimately leads to green budget receipts and expenditures that inhibit the role of economic development in improving the environmental environment [25]. On the contrary, the government will not only increase the cost of enterprises through green fiscal revenue and constrain enterprises’ pollutant-discharge behavior but also encourage enterprises to invest in environmental protection and improve technology through green fiscal expenditure measures. As a result, reducing pollution emissions and making the EKC inflection point could come earlier [22,26].

### 2.3. Eco-Environmental Effects of Fiscal Ecological Compensation

Ecological compensation is an institutional arrangement that adjusts the interest relationship between ecological beneficiaries and ecological protectors through a market-oriented mechanism to realize the internalization of ecological protection externalities [27]. At present, due to the absence of relevant legal systems and property rights arrangements, government-led vertical financial transfers have become an important means of ecological compensation. In other words, fiscal ecological compensation is the most important expression of ecological compensation [6].

The policy practice of fiscal eco-compensation could be traced back to the value-added tax-based tax-sharing system of the Brazilian State of Parana in 1992. This scheme has a positive effect on the ecological protection of the State of Parana [28]. Since then, Ecological Value-Added Tax and Service Tax has been used as a benchmark by other regions and countries and has created a positive incentive for environmental governance [29]. China’s fiscal ecological compensation system can be traced back to the returning farmland to the forest system in the 1990s. However, the formal fiscal ecological compensation system is the national key ecological function area transfer payment system, which was piloted and implemented in 2008. Similarly, due to the unclear subjects responsible for ecological environmental governance in China and the immature market mechanism, vertical ecological transfer payments that incorporate ecological protection indicators into the intergovernmental financial redistribution standards have become the main means of ecological compensation [6]. In Chinese practice, fiscal ecological compensation has played an important positive role in boosting the governance behavior of the local government’s ecological environment through the effect of financial compensation. It also significantly improves the local ecological environment and the level of supply of public services for the livelihoods of populations [30,31,32,33,34].

According to the above research, ecological financial compensation has become an important means for the government to coordinate environmental governance among regions. However, the above studies not only fail to reach a consensus on China’s green development path but also have not yet clarified the theoretical basis, influence, and mechanism of the realization path of ecological financial compensation to promote green development. In this respect, this paper attempts to complement the inadequacy of the above research by incorporating economic development and the ecological environment into the same analytical framework.

## 3. Empirical Design

### 3.1. Data Sources

Geographical data, such as topographic reliefs, were calculated based on Feng et al. [35]. Currently, there are two kinds of time-series luminous remote sensing data commonly used: Defense Meteorological Satellite Program (DMSP)/Operational Linescan System (OLS) and Visible Infrared Imaging Radiometer Suite (NPP)/VIIRS (Visible Infrared Imaging Radiometer Suit). The first kind was adopted by us. Other data came from The Bulletin on China’s Ecological and Environmental Conditions and the Statistical Yearbook of Chinese Cities, and the authors have carried on the corresponding manual statistical collation.

The data used in this paper come from the dataset of county-level regions in China in 2017 and 2018. Among them, the ecological environment status index and its five sub-indicators are the main explained variables, and the data come from the 2017 and 2018 “China Ecological Environment Status Bulletin”. The transfer payment data for the national key ecological function areas are obtained by applying to the Ministry of Finance for disclosure. Economic, social, and geographic data, such as Gross Domestic Product (GDP) per capita, number of industrial enterprises above designated size, population density, proportion of secondary industry, administrative area, average altitude, etc., are derived from public statistics such as the “China Urban Statistical Yearbook” in 2018 and 2019. Geographic data, such as terrain relief, are calculated according to the research of Feng et al. [35]. Light-intensity data includes the DMSP/OLS radiometric calibrated nighttime light data products for 2017 and 2018 as well as the stable light data products.

### 3.2. Variable Description

#### 3.2.1. Explained Variables

Ecological index (*EI*) is the explained variable in this paper, and its value represents the overall ecological environment quality: the larger the *EI*, the better the ecological environment quality; the smaller the *EI*, the worse the ecological environment quality. The specific calculation formula of *EI* is as follows:

EI = 0.25 × BAI + 0.2 × VCI + 0.2 × WNDI + 0.2 × LSI + 0.15 × PLI

Among them, *BAI* refers to biological abundance index. *VCI* refers to vegetation coverage index. *WNDI* refers to water network density index. *LSI* refers to land stress index. *PLI* refers to the pollution load index. Indicators are selected from the Environmental Protection Department of the People’s Republic of China issued by the state environmental protection standards in 2015. The calculation method for ecological environment in it is to weight these five indicators accordingly. This method was also adopted in this paper. Combined with the availability of data in each county and district, these five items were selected in this study as the explanatory variables for the indicators of ecological environment status.

In the empirical research, in order to test the robustness of the empirical results and analyze the similarities and differences between different ecological environment indicators, this paper uses the ecological environment status index and its five sub-indicators to conduct subsequent empirical research.

#### 3.2.2. Core Explanatory Variables

Per capita GDP is one of the core explanatory variables of this paper, which is used to represent the current level of economic development. At the same time, in order to truly reflect the economic conditions of the evaluated regions, this paper deflates nominal GDP using 2017 as the base period to avoid the impact of rising price indices.

The national key ecological function area transfer payment (hereinafter referred to as ecological transfer payment) is another core explanatory variable of this paper. As it is not possible to obtain specific information on the size of ecological transfer funds received by each county-level region, this paper uses the list of county-level regions receiving ecological transfers to set dummy variables. Specifically, the county-level region that has obtained ecological transfer payment takes the value of 1, and otherwise, it is 0.

#### 3.2.3. Control Variables

The ecological environment of an area at county level is affected not only by the level of economic development but also by other economic, social, and geographic factors [6,19]. Therefore, in order to control for problems caused by omitted variables, this paper introduces control variables into the empirical model to control for the effects of other factors on the state of the ecosystem. This includes the number of industrial enterprise units above a certain size, population density, the proportion of secondary industry, the area of the administrative area, the average altitude and the topographic relief. Among them, the number of industrial enterprises above designated size is represented by the number of large-scale enterprises in the county-level area. Population density is expressed in terms of population per square kilometer. The descriptive statistics of the above variables are shown in Table 1.

### 3.3. Model Building

#### 3.3.1. The Relationship between Economic Development and Ecological Environment

As stated earlier, there may be a non-linear relationship between economic development and the ecological environment. However, the specific form of the non-linear relationship is not clear. Different forms of non-linear relationships may be associated with differences in the selection of sample data and proxy variables. There is a lack of research at the county level, which is the basis of overall economic and social governance in China, and in particular, the possible impact and mechanisms of action of fiscal eco-compensation are not clear. Therefore, based on the data from county-level regions in China, this paper first examines the relationship between economic development and the ecological environment.

Referring to the research of Wu et al. [36], this paper uses the stepwise regression method to first test the relationship between economic development and ecological environment and then test the validity of the EKC hypothesis. The model is set as follows:

(1) $$\ln EI_{i}\;=\;\alpha_{0}\;+\;\beta_{1}\ln pcgdp_{i}\;+\;\beta_{2}{\left(\ln pcgdp_{i}\right)}^{2}\;+\;\beta_{3}{\left(\ln pcgdp_{i}\right)}^{3}\;+\;\beta_{4}fec_{i}\;+\;\lambda^{\prime }X_{i}\;+\;\varepsilon_{i}$$

Among them, *EI_i_* represents the ecological environment status index of county-level region; *i*. *pcgdp_i_* represents the per capita GDP of county-level region; and *i*. *fec_i_* is the dummy variable of whether county-level region *i* has obtained the transfer payment for national key ecological function zones. If the transfer payment is obtained, then *fec_i_* = 1; otherwise, *fec_i_* = 0. *X_i_* represents a column vector composed of a series of other control variables. $\varepsilon_{i}$ is a random disturbance term. *pcgdp_i_* is one of the important variables affecting the ecological environment. As already mentioned in Section 1, there may be a nonlinear relationship between the economic growth and ecological environment. The specific form of the nonlinear relationship is uncertain. Therefore, the variables in model (1) are set to the first-power, second-power, and third-power items of *pcgdp_i_*, respectively. Among them, *β*_1_*–β*_3_ are the core explanatory variable parameters concerned in this paper, and the validity of the ecological EKC hypothesis can be identified by the positive, negative, and significance of its coefficients. Firstly, when *β*_1_*–β*_3_ are not significant, economic development is not related to the ecological environment. Secondly, when *β*_2_*–β*_3_ is not significant, but *β*_1_ is significant and greater than zero, economic development and ecological environment are positively correlated. In the contrast, when *β*_1_ is significant and less than zero, the two are negatively correlated. Thirdly, when *β*_3_ is not significant, but *β*_2_ is significant and greater than zero, the two are in a positive U-shaped relationship consistent with the ecological EKC hypothesis. On the contrary, when *β*_2_ is significant and less than zero, the two have an inverted U-shaped relationship. Finally, when *β*_3_ is significant and greater than zero, the two have an N-shaped relationship. Conversely, when *β*_3_ is significant and less than zero, the two are in an inverted N-shaped relationship.

#### 3.3.2. The Impact of Fiscal Ecological Compensation

In order to further analyze the impact of fiscal ecological compensation on economic green development, referring to the research of He and Wang, and Antweiler [23,37], this paper further adds the interaction term of the product term of *fec_i_* and *pcgdp_i_* on the basis of model (1). The specific form of the model (2) is as follows:

(2) $$\begin{array}{l} \ln EI_{i}\;=\;\alpha_{0}\;+\;\beta_{1}\ln pcgdp_{i}\;+\;\beta_{2}{\left(\ln pcgdp_{i}\right)}^{2}\;+\;\beta_{3}{\left(\ln pcgdp_{i}\right)}^{3}\\ \;+\;\beta_{4}fec_{i}\;+\;\gamma \ln pcgdp_{i}\times fec_{i}\;+\;\lambda^{\prime }X_{i}\;+\;\varepsilon_{i} \end{array}$$

Among them, this paper uses the significance, magnitude, and direction of *β*_1_*–β*_3_ and *λ* to judge the impact of fiscal ecological compensation on the green development path. Based on the meaning, magnitude, and direction of γ, we judge whether the impact of *pcgdp_i_* and *fec_i_* on the green development path is the same. Further, we determine whether the impact of national key ecological function area transfer payment on *pcgdp_i_* is inhibited or promoted.

## 4. Empirical Analysis

### 4.1. Basic Regression

This paper uses the least squares method to estimate model (1) and controls the annual trend and the fixed effects of county-level regions. At the same time, it also adopts robust heteroscedasticity as a standard error to control the heteroscedasticity problem. Table 2 illustrates the empirical results of the basic regression.

On the basis of stepwise regression testing the relationship between economic development and ecological environment, model (5) is the estimation for the whole sample. Models (6) and (7) were estimated for the 2017 and 2018 subsamples, respectively, with the aim of testing whether there are temporal differences in the ecological EKC assumptions. Without adding any control variables, from the estimation results of model (1) to model (3), *R*^2^ gradually increases, and the fitting degree of the model gradually improves. Among them, the coefficient of the cubic term of per capita GDP in model (3) is significant at the 1% significance level, and the model has a higher degree of fit. Therefore, this paper retains the cubic term of per capita GDP in model (4) on the basis of added ecological transfer payment. Model (4) demonstrates that the ecological transfer payment has a positive effect on the ecological environment at the 1% significance level. In order to accurately estimate the parameters of the model and avoid the problem of estimation bias caused by missing variables, model (5) further adds other control variables on the basis of model (4). From the regression results of model (5), the fitting degree of the model is further improved. The coefficient of the cubic term of per capita GDP is positive at the 1% significance level, indicating that there is an N-shaped nonlinear relationship between economic development and the ecological environment. The inflection points of the curve are CNY 23,195 and CNY 276,952 respectively.

The critical point calculation formula of N-shaped curve is as follows:

$$\frac{\partial \ln EI_{i}}{\partial \ln pcgdp_{i}}\;=\;\beta_{1}\;+\;2\beta_{2}\ln pcgdp_{i}\;+\;3\beta_{3}{\left(\ln pcgdp_{i}\right)}^{2}$$

Set it to zero: $\beta_{1}\;+\;2\beta_{2}\ln pcgdp_{i}\;+\;3\beta_{3}{\left(\ln pcgdp_{i}\right)}^{2}\;=\;0\;$.

To solve this equation: $\ln pcgdp_{i}\;=\;\frac{-2\beta_{2}\;\pm \;\sqrt{4\beta_{2}{}^{2}\;-\;12\beta_{1}\beta_{3}}}{6\beta_{3}}$. Finally, the value of *pcgdp_i_* was inversely solved by ln*pcgdp_i_*.

This means that when GDP per capita is between CNY 23,195 and CNY 276,952, economic development will gradually deteriorate the ecological environment; when GDP per capita is higher than CNY 276,952, economic development will bring about ecological improvement. The dummy variable coefficient of ecological transfer payment is positive at the 1% significance level, indicating that fiscal ecological compensation may significantly improve the ecological environment. As in the Brazil and India studies, the increase of fiscal ecological compensation has a significant positive effect on environmental improvement [28,29]. The regression results of the two subsamples in 2017 and 2018 show that the coefficients of the cubic terms of per capita GDP are all positive at the 1% significance level, which is consistent with the estimation results of the full sample. This means the N-shaped non-linear relationship between economic development and the ecological environment persists. The inflection points of the sample nonlinear relationship in 2017 were CNY 22,663 and CNY 299,628, and the inflection points of the nonlinear relationship in 2018 were CNY 21,498 and CNY 299,659, respectively. The conclusions of the sub-sample study are basically the same as that of the whole-sample study. Taking the 1444 samples in 2018 as an example, there are 304 counties with per capita GDP below CNY 21,498, accounting for 21.05% of the total sample. This shows that this region is in the first stage of an N-shaped nonlinear relationship, and economic development will bring ecological environmental improvement. Furthermore, there are 1135 counties with per capita GDP between CNY 21,498 and CNY 299,659, accounting for 78.60% of the total sample. That is, the region is in the second stage of the N-shaped nonlinear relationship, and economic development will gradually deteriorate the ecological environment. Furthermore, the only counties with per capita GDP higher than CNY 299,659 are Otuoke Banner, Wushen Banner, Yijinhuoluo Banner, Kunshan City, and Yiwu County, accounting for only 0.35% of the total sample. That is, the regions enter the third stage of the N-shaped non-linear relationship, where economic development will improve the ecological situation.

The N-shaped nonlinear relationship between economic development and ecological environment shows that, along with economic development, the ecological environment goes through three stages of improvement, deterioration, and improvement in turn. In the first stage, due to the relatively slow economic development, the speed of ecological environment governance and restoration is greater than the speed of consumption and destruction brought about by economic development. However, at the second stage, economic development has reached a certain scale. The “Extensive” development model has enabled China to achieve economic development only through increased consumption of factors of production, including natural resources, resulting in a rate of resource consumption that far exceeds the rate of resource regeneration. As a result, the negative impact of economic development on the ecological environment becomes apparent. In addition, the industrial shift from the east to the central and western parts of the country in recent years has also led to a continuous decline in environmental conditions, so there is a negative correlation with it [38,39]. Studies have shown that ultimately improving the environmental environment depends on public demand for a high-quality environmental environment in a highly developed economy [7,23,24]. A positive correlation between ecological environment quality and income level exists only when per capita income exceeds a certain level. In other words, people’s demand for ecological environment quality will increase with the increase of income. The objective conditions for a change in the pattern of economic development are also in place. Inflection point in the N-shaped non-linear relationship between economic development and ecology emerges. Society has entered a phase where economic growth and ecological environment are in harmony.

With respect to control variables, the empirical results of this article are essentially consistent with existing studies [7,21]. Specifically, the number of industrial enterprises above designated size, population density, and terrain fluctuation have a positive impact on the ecological environment at the significance level of 10%, 5%, and 1%, respectively. Altitude has a negative impact on the ecological environment at the 10%, 1%, and 1% significance levels, respectively.

### 4.2. Analysis of Sub-Indicators of Ecological Environment Condition Index

According to the reliability of the test results, this paper uses the five sub-indices of the ecological environment condition index to replace the ecological environment condition index as the explained variables to test the robustness. The corresponding empirical results are shown in the model in Table 3 (1)–(5).

As shown in Table 3, for different sub-indices of the ecological environment condition index, there is a certain difference in the nonlinear relationship between it and per capita GDP. That is, there are different forms of non-linear relationships between the different sub-indicators of the economic development and ecological status index. Specifically, the cubic coefficient of per capita GDP of the biological abundance index in model (1) is positive at the 1% significance level. It is showed that there is an N-shaped nonlinear relationship between the biological abundance index and economic development. The quadratic coefficients of GDP per capita for the vegetation cover index and the water network density index in models (2) and (3) are both negative at the 1% significance level. It is illustrated that an inverted U-shaped non-linear relationship exists between vegetation cover index and water network density index and economic development. The coefficient of the cubic term of GDP per capita for the land stress index in model (4) is negative at the 1% significance level. This means there is an inverted N relationship between the land stress index and economic development. The quadratic coefficient of per capita GDP of the pollution load index in model (5) is negative at the 1% significance level. This means there is an inverted U-shaped nonlinear relationship between the pollution load index and economic development. The above findings are consistent with traditional EKC theory [7,23].

On the whole, there is an intricate relationship between economic development and the ecological environment. The nonlinear relationship between the two verified in this paper may be real. Since the meanings of the various sub-indicators differ, there are differences in their non-linear forms in the sample interval.

### 4.3. Endogenous Analysis

In order to solve the endogenous problem caused by the two-way causal relationship between economic development and ecological environment, this paper attempts to use the instrumental variable method to discuss this endogenous problem in the model. In this paper, the nighttime light intensity (DMSP/OLS) and the per capita GDP (L.ln*pcgdp*) of the county-level area are used as instrumental variables. The 2SLS method is adopted for empirical research. The empirical results are shown in Table 4. When only the light intensity at night is used as an instrumental variable, the *F*-value of the first-stage regression is greater than 10, indicating that there is no weak instrumental variable problem. At the same time, the cubic coefficient of per capita GDP is positive at the 1% significance level. This means the N-shaped nonlinear relationship between economic development and ecological environment still exists significantly, and the basic regression results are more credible. Finally, when using only lagged one-period GDP per capita as an instrumental variable and taking the two variables as instrumental variables into the estimation equation, the latter fails to pass the over-identification test. Therefore, the *F* values of the above two equations in the one-stage regression are all greater than 10, and it could be observed that there is an N-shaped nonlinear relationship between the level of economic development and the ecological environment. This result further confirms the credibility of the aforementioned basic regression results.

### 4.4. The Impact of Fiscal Ecological Compensation

Table 5 shows the effect of the model’s fiscal ecological compensation (2) on the relationship between economic development and the ecological environment. Among them, model (1) uses the ecological environmental condition index as a dependent variable to explore the impact of fiscal ecological compensation. At the same time, in order to ensure the robustness of the research results, the models (2)–(6) are, respectively, based on the biological abundance index, vegetation coverage index, water network density index, land stress index, and pollution load index, which are the five sub-indexes of the ecological environment condition index. Indicators are used as dependent variables to explore the impact of fiscal ecological compensation. It could be seen from Table 5 that after adding the interaction term to the basic model, the nonlinear correlation between various ecological environment indicators and economic development is still significant, which is consistent with the nonlinear correlation obtained by the basic regression. In models (1)–(4), the coefficients of fiscal ecological compensation are all significantly positive. It is indicated that fiscal ecological compensation can directly improve the ecological environment. The coefficient of interaction between economic development variables and fiscal ecological compensation variables is significant. As a result, it could be inferred that fiscal ecological compensation could affect the relationship between economic development and ecological environment, thereby indirectly affecting the ecological environment.

**Figure 1:**
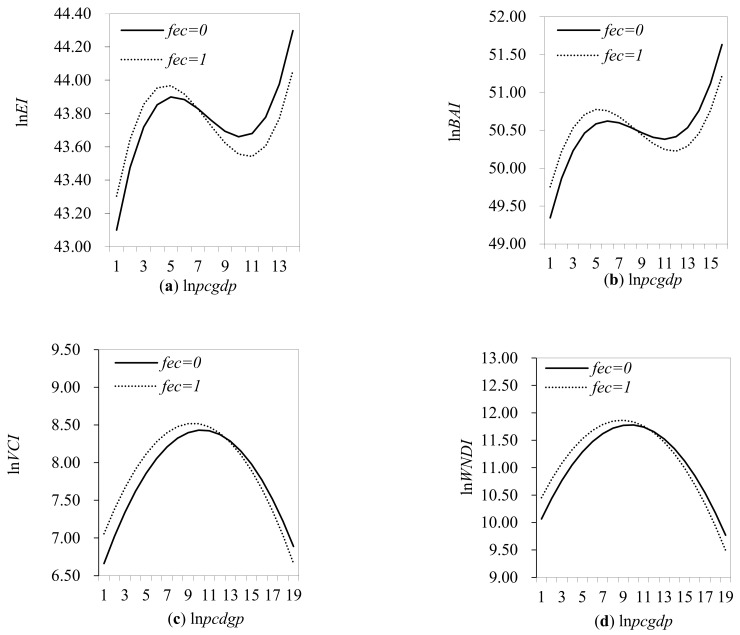

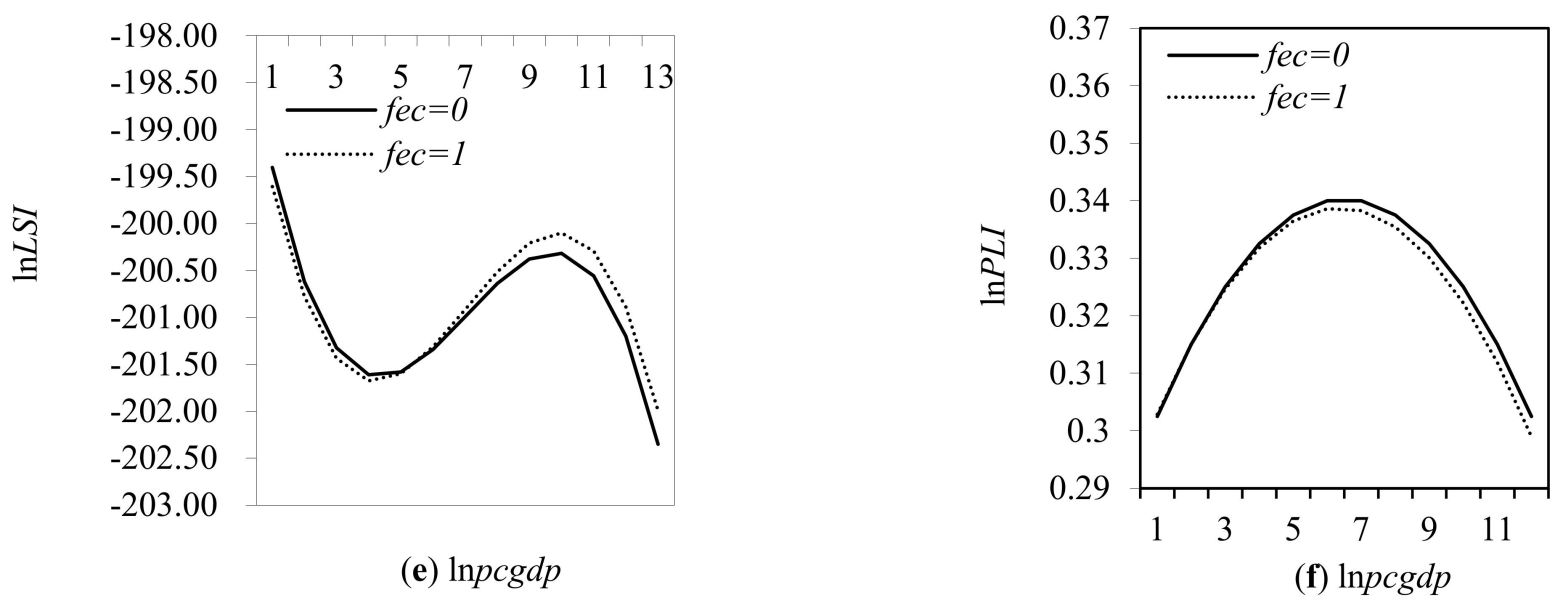
Impact of financial eco-compensation.

To visualize the indirect effects of fiscal ecological compensation on the relationship between economic development and ecological environment, Figure 1 shows the economic development and ecological environment in areas without fiscal ecological compensation (*fec* = 0) and with fiscal ecological compensation (*fec* = 1). Figure 1a presents the impact of fiscal eco-compensation on the relationship between the ecosystem condition index and economic development. It could be seen that fiscal ecological compensation has led to a change in the non-linear relationship between economic development and the ecological status index. The specific performance is as follows: Fiscal ecological compensation shifts the non-linear curve between economic development and the index of ecological status upwards in the interval of GDP per capita below CNY 58,747. However, when the per capita GDP is higher than CNY 58,747, the fiscal ecological compensation makes the nonlinear curve between the economic development and the ecological environment condition index move downward. It could be indicated that the fiscal ecological compensation has changed the impact of economic development on the ecological environment condition index. In the full sample, approximately 79.68% of the counties are in the interval where GDP per capita is below CNY 58,747, i.e., to the left of the intersection of the curves. The reason for this may be that in the early stages of economic development, fiscal ecological compensation can fully compensate for the opportunity costs of development that regions sacrifice by forgoing economic development. Therefore, local governments focus on protecting the ecological environment. However, with economic development, fiscal ecological compensation is gradually insufficient to make up for the development opportunity cost sacrificed by local governments for giving up economic development. In this case, local governments prefer to sacrifice the ecological environment in exchange for “extensive” economic development. According to the existing research, whether fiscal ecological compensation can encourage local governments to protect the environment depends on whether they can increase local financial expenditure to improve ecological and environmental conditions under China’s environmental governance [40].

The regression results for the sub-indices of the ecological environment condition index further prove the robustness of this result. Figure 1b illustrates the impact of fiscal ecological compensation on the relationship between the organic abundance index and economic development. It can be seen that fiscal ecological compensation changes the nonlinear relationship between biological abundance index and economic development, which is embodied as follows: When GDP per capita is below CNY 126,696, fiscal ecological compensation shifts the nonlinear curve between the biological abundance index and economic development upward. When GDP per capita is higher than CNY 126,696, fiscal ecological compensation shifts the nonlinear curve between the index of biological abundance and economic development downward. Clearly, the results conform to Figure 1a, which confirms the economic logic of Figure 1a. Figure 1c presents the impact of fiscal ecological compensation on the relationship between vegetation cover index and economic development. In the range where the per capita GDP is lower than CNY 76,826, the fiscal ecological compensation makes the nonlinear relationship between economic development and the vegetation cover index move upward. When GDP per capita is higher than CNY 76,826, fiscal ecological compensation then shifts the curve of the non-linear relationship between economic development and vegetation cover index downwards. Figure 1d shows how fiscal ecological compensation affects the relationship between the water system density index and economic development. Similarly, in the interval where GDP per capita is below CNY 209,546, fiscal ecological compensation shifts the curve of the non-linear relationship between economic development and the water network density index upwards. On the contrast, when GDP per capita is higher than CNY 209,546, fiscal ecological compensation shifts the curve of the non-linear relationship between economic development and the water network density index downwards. Figure 1c,d both present the same results. At low levels of economic development, fiscal eco-compensation can significantly improve the ecological environment. However, as the level of economic development increases, fiscal eco-compensation exacerbates the negative impact of economic development on the ecological environment. This result is different from the specific conclusions in Figure 1a,b. The reason for this may lie in the difference between the initial non-linear relationship between economic development and the vegetation cover index and the water network density index and the initial non-linear relationship between economic development and the ecological condition index and the biological abundance index. However, the following consistent results can still be drawn. When the level of economic development is low, fiscal ecological compensation could significantly improve the degree of improvement of the ecological environment caused by economic development. However, as the level of economic development increases, fiscal ecological compensation has a negative impact on the relationship between economic development and the ecological environment. Figure 1e presents the impact of fiscal ecological compensation on the relationship between land stress index and economic development. When the per capita GDP is lower than CNY 43,840, the fiscal ecological compensation makes the nonlinear relationship between economic development and the land coercion index move downward. When GDP per capita is higher than CNY 43,840, fiscal ecological compensation shifts the non-linear relationship between economic development and the land stress index upwards. While this outcome differs from Figure 1a,b, the reason may be that the meanings of specific environmental indicators are different. One possible reason is the contradiction between the unlimited demand for rapid economic growth and the limitation of land supply. Although this result is not significant, the result is still consistent with the previous analysis. Figure 1f presents the impact of fiscal ecological compensation on the relationship between the pollution load index and economic development. It could be seen that with economic development, fiscal ecological compensation will slow down the adverse impact of economic development on the ecological environment. However, this negative effect is found not to be statistically significant and there is only a slight economic difference (only −0.0007).

Overall, fiscal ecological compensation has changed the nonlinear correlation between economic development and ecological environment. Fiscal ecological compensation can be directly used to improve the ecological environment. On the other hand, fiscal ecological compensation can fully compensate for the development opportunity cost sacrificed by the region because of giving up economic development. This policy may therefore encourage the government to pay greater attention to improving the ecological environment. Fiscal ecological compensation can significantly improve the degree of improvement of the ecological environment caused by economic development when the level of economic development is low. The robustness of this result is further proved from the test of the five sub-indices of the ecological environment condition index.

## 5. Research Conclusions and Policy Recommendations

### 5.1. Research Conclusions

In the context of green development, this paper uses the data of China’s county-level regions in 2017 and 2018 to construct an econometric model to analyze the impact of fiscal ecological compensation on China’s green development path. The main conclusions of this study are as follows:

Firstly, there is an N-shaped, nonlinear correlation between China’s economic development and the ecological environment. This means that, in parallel with economic development, the ecological environment goes through the three stages of improvement, degradation, and improvement. In the first stage, due to the relatively slow economic development, the speed of ecological environment governance and restoration is greater than the speed of consumption and destruction brought about by economic development. However, under the “extensive” development model, China could only achieve economic growth by increasing the consumption of production factors, including natural resources, resulting in the speed of resource consumption far exceeding the speed of resource regeneration. As a result, the first inflection point appears. This means that the detrimental effects of economic development on the ecological environment arise. Only when per capita income exceeds a certain level, people’s demand for eco-environmental quality will increase with the growth of income. The objective conditions for a change in the pattern of economic development are also in place. Inflection point in the N-shaped non-linear relationship between economic development and ecology emerges. Society has entered a phase where economic growth and ecological environment are in harmony.

Secondly, fiscal ecological compensation has a direct positive impact on ecological environment governance. On the one hand, fiscal ecological compensation has curbed the ecological environment damage caused by the “extensive” development model of areas with important ecological functions, such as key ecological counties, prohibited development areas, and national ecological civilization experimental areas. On the other hand, fiscal ecological compensation provides direct public financial support for the above-mentioned areas to protect the ecological environment and provide basic public services, which could significantly improve the local ecological environment.

Finally, fiscal ecological compensation has an indirect impact on ecological environment governance by affecting economic development. For regions with a low level of economic development, fiscal ecological compensation may essentially offset the development opportunity cost because of policy requirements. For this reason, the regions benefiting from this policy are at an earlier stage of the coordinated development of economic development and the ecological environment. For areas with a high level of economic development, fiscal ecological compensation will limit the economic development of the area and place the area in a stage of negative correlation between economic development and ecological environment. The reason is that the opportunity cost for local governments to give up development due to the protection of the ecological environment is high. Due to insufficient fiscal ecological compensation, local governments still adopt an “extensive” development model to some extent.

### 5.2. Policy Recommendations

Firstly, transforming the economic development model and delivering high-quality economic development is a high priority. At present, about 78.60% of county-level areas in China are in the “green development trap” stage. In order to get rid of the shackles of the “green development trap” and achieve high-quality economic development, the most fundamental thing is to start from economic development itself and change the existing “extensive” economic development model. The high-quality development of the economy requires that the output of economic activities should be increased under the condition that the input scale of production factors is certain. This means the output efficiency of economic activities should be improved. In this way, the excessive consumption of natural resources by economic development under the “rough and tumble” development model could be transformed, and green economic development could ultimately be achieved.

Secondly, we recommend expanding the coverage of fiscal ecological compensation and deepening the design of the fiscal ecological compensation system. Fiscal ecological compensation can directly affect the governance of the ecological environment. However, according to the current situation in China, the coverage of fiscal ecological compensation is small, which makes it unable to give full play to its due ecological environment governance effect. Therefore, in order to give full play to the ecological environment governance effect of fiscal ecological compensation, the government may consider the following suggestions. On the one hand, the government should appropriately expand the coverage of fiscal ecological compensation. This means the government should gradually include more counties in the fiscal eco-compensation policy, with an aim to bring to bear the positive impact of fiscal eco-compensation in eco-environmental management on a national scale. On the other hand, the government should continuously optimize the institutional design of financial eco-compensation to provide stable and continuous public funding support for it. Thereby, this is conducive to deepening the ecological governance effect of fiscal ecological compensation and helping to achieve green development.

Thirdly, focus should be placed on systematic evaluation of the policy effects of fiscal ecological compensation. Fiscal ecological compensation policy could significantly improve the ecological environment in areas with low economic development level in the short term. However, in the long term, this will hinder the economic transformation and development of regions with a high level of economic development. Fiscal ecological compensation should implement differentiated fund scale allocation on the basis of fully considering the level of local economic development. In addition to “adjusting measures to local conditions”, fiscal ecological compensation should also be “growing right crop for right time”. Therefore, the government should systematically evaluate the policy effects of fiscal ecological compensation. It should not only pay attention to the improvement role of fiscal ecological compensation in green development in the short and medium term but also pay attention to its impact on local economic development. This avoids disrupting the economic development model in such a way that the negative effects become apparent in the long term, making it difficult to achieve green development at a local level.

There is the possibility and necessity of further research on the research topic of this paper. For example, how to incorporate the spillover effect between regions into the analysis framework will be a very interesting research topic. Solving this problem will be definitely help coordinate the green and sustainable development among jurisdictions. Therefore, the positive impact of fiscal ecological compensation will be further released.

## Figures and Tables

**Table 1:** Descriptive statistics of each variable.

Variables	Sign	Unit	Obs	Average	Standard Deviation	Min	Max
Ecological Condition Index	*EI*	%	2894	61.31	15.58	16.00	91.20
Biological Abundance Index	*BAI*	%	2894	44.19	21.58	3.20	96.80
Vegetation Cover Index	*VCI*	%	2894	81.36	17.10	8.30	100.00
Water Network Density Index	*WNDI*	%	2894	35.30	22.72	0.60	100.00
Land Stress Index	*LSI*	%	2894	14.99	16.75	0.20	100.00
Pollution Load Index	*PLI*	%	2894	98.35	3.79	49.90	100.00
Whether Receiving an Ecological Transfer	*fec*	-	2894	0.228	0.419	0	1
GDP Per Capita	*pcgdp*	CNY	2894	45,000	42,000	5245	450,000
Number of Industrial Enterprise Units above Scale	*nied*		2836	140	211	1	2499
Share of Secondary Sector	*Ind2*	%	2894	41.34	13.85	4.84	86.44
Population Density	*pd*	million people/km^2^	2894	0.04	0.03	0	0.44
Administrative Area	*area*	km^2^	2894	3189.83	6803.10	59.00	110,000
Elevation Mean	*altitude*	m	2894	712.10	906.67	−0.97	5113.84
Terrain Undulation	*slope*	m	2894	0.93	1.15	0	6.44
Light Intensity	*li*	10,000	2894	0.77	1.67	0	26.55

**Table 2:** Basic regression empirical results of the relationship between economic development and ecological environment.

	(1)	(2)	(3)	(4)	(5)	(6)	(7)
ln*pcgdp*	−0.0560 ***	0.9803 ***	7.0480 ***	7.4185 ***	10.5810 ***	10.8884 ***	10.3028 ***
	(−6.39)	(6.26)	(3.44)	(3.63)	(5.65)	(4.04)	(3.96)
(ln*pcgdp*)^2^		−0.0486 ***	−0.6173 ***	−0.6472 ***	−0.9485 ***	−0.9746 ***	−0.9249 ***
		(−6.62)	(−3.21)	(−3.38)	(−5.40)	(−3.86)	(−3.79)
(ln*pcgdp*)^3^			0.0177 ***	0.0185 ***	0.0280 ***	0.0287 ***	0.0273 ***
			(2.95)	(3.10)	(5.12)	(3.65)	(3.59)
*fec*				0.0929 ***	0.0504 ***	0.0504 **	0.0506 ***
				(6.47)	(3.69)	(2.52)	(2.70)
ln*nied*					0.0200 *	0.0176	0.0222
					(1.95)	(1.21)	(1.53)
ln*pd*					0.0311 **	0.0345	0.0278
					(2.09)	(1.62)	(1.33)
ln*Ind2*					−0.0016 ***	−0.0018 ***	−0.0014 **
					(−3.28)	(−2.59)	(−2.04)
ln*area*					−0.0251 *	−0.0207	−0.0293
					(−1.84)	(−1.08)	(−1.51)
ln*altitude*					−0.5060 ***	−0.5118 ***	−0.5001 ***
					(−17.02)	(−12.04)	(−11.98)
ln*slope*					0.5191 ***	0.5234 ***	0.5147 ***
					(19.27)	(13.58)	(13.61)
*cons*	−14.9847	−19.0095	−40.4708 *	−36.8851	−44.6904 **	−32.3585 ***	−30.1173 ***
	(−0.69)	(−0.89)	(−1.78)	(−1.64)	(−2.30)	(−3.41)	(−3.30)
Annual Trends	YES	YES	YES	YES	YES	NO	NO
District Fixed Effects	YES	YES	YES	YES	YES	YES	YES
*N*	2894	2894	2894	2894	2832	1427	1405
*F*	25.5942	29.8285	24.8436	28.1095	110.3445	59.4002	59.9515
*R* ^2^ *_adj*	0.0465	0.0583	0.0601	0.0759	0.3326	0.3248	0.3356
inflection point	-	-	-	-	CNY 23,195	CNY 22,663	CNY 21,498
	-	-	-	-	CNY 276,952	CNY 299,629	CNY 299,659

Notes: *T*-statistics are in parentheses. ***, **, and * indicate significance at the 1%, 5%, and 10% levels, respectively.

**Table 3:** The empirical results of the relationship between economic development and the sub-indicators of the ecological environment condition index.

	(1) *BAI*	(2) *VCI*	(3) *WNDI*	(4) *LSI*	(5) *PLI*
ln*pcgdp*	11.1273 ***	1.4550 ***	1.8587 ***	−51.9992 ***	0.0803 *
	(3.23)	(8.31)	(5.34)	(−8.80)	(1.93)
(ln*pcgdp*)^2^	−0.9571 ***	−0.0726 ***	−0.0820 ***	4.5749 ***	−0.0049 **
	(−2.94)	(−8.89)	(−5.03)	(8.29)	(−2.42)
(ln*pcgdp*)^3^	0.0271 ***			−0.1323 ***	
	(2.65)			(−7.71)	
*fec*	0.1655 ***	0.0696 ***	0.1494 ***	−0.0419	−0.0034 *
	(7.06)	(4.87)	(5.80)	(−0.79)	(−1.95)
*cons*	−19.3189	−16.3955	19.7236	152.3289 **	1.7395
	(−0.58)	(−0.92)	(0.54)	(2.18)	(0.55)
Control	YES	YES	YES	YES	YES
Annual Trends	YES	YES	YES	YES	YES
District Fixed Effects	YES	YES	YES	YES	YES
*N*	2832	2832	2832	2832	2832
*F*	212.9734	63.6005	187.3018	57.4357	18.2326
*R* ^2^ *_adj*	0.4257	0.4107	0.5692	0.1781	0.1478

Notes: *T*-statistics are in parentheses. ***, **, and * indicate significance at the 1%, 5%, and 10% levels, respectively.

**Table 4:** Empirical results of endogenous analysis of the relationship between economic development and ecological environment.

	*DMSP/OLS*	L.ln*pcgdp*	*DMSP/OLS* and L.ln*pcgdp*
ln*pcgdp*	97.4864 ***	11.1606 ***	11.5878 ***
	(2.85)	(5.63)	(5.80)
(ln*pcgdp*)^2^	−9.1336 ***	−1.0010 ***	−1.0429 ***
	(−2.91)	(−5.40)	(−5.58)
(ln*pcgdp*)^3^	0.2832 ***	0.0296 ***	0.0309 ***
	(2.95)	(5.13)	(5.32)
*fec*	0.0386 *	0.0501 ***	0.0491 ***
	(1.73)	(3.65)	(3.59)
*cons*	−381.4152 ***	−45.9006 **	−48.5020 **
	(−3.31)	(−2.33)	(−2.46)
Control	YES	YES	YES
Annual Trends	YES	YES	YES
District Fixed Effects	YES	YES	YES
*N*	2832	2805	2805
*R* ^2^ *_adj*	-	0.3344	0.3342

Notes: *T*-statistics are in parentheses. ***, **, and * indicate significance at the 1%, 5%, and 10% levels, respectively.

**Table 5:** The regression results of the impact of fiscal ecological compensation.

	(1) *EI*	(W) *BAI*	(E) *VCI*	(4) *WNDI*	(5) *LSI*	(5) *PLI*
ln*pcgdp*	11.8659 ***	13.1744 ***	1.6611 ***	2.0811 ***	−53.7592 ***	0.0825 *
	(6.01)	(3.57)	(8.36)	(5.48)	(−8.44)	(1.82)
(ln*pcgdp*)^2^	−1.0586 ***	−1.1326 ***	−0.0818 ***	−0.0919 ***	4.7258 ***	−0.0050 **
	(−5.76)	(−3.29)	(−8.88)	(−5.20)	(8.01)	(−2.29)
(ln*pcgdp*)^3^	0.0311 ***	0.0321 ***			−0.1366 ***	
	(5.47)	(2.99)			(−7.51)	
ln*pcgdp* × *fec*	−0.0686 ***	−0.1094 **	−0.0686 **	−0.0740 *	0.0940	−0.0007
	(−2.73)	(−2.28)	(−2.44)	(−1.83)	(1.02)	(−0.16)
*fec*	0.7533 ***	1.2854 ***	0.7717 ***	0.9067 **	−1.0047	0.0042
	(2.96)	(2.63)	(2.71)	(2.21)	(−1.06)	(0.09)
*cons*	−50.3490 **	−28.3344	−18.2660	17.7061	160.0797 **	1.7193
	(−2.57)	(−0.85)	(−1.03)	(0.49)	(2.26)	(0.54)
Control Variables	YES	YES	YES	YES	YES	YES
Year Trend	YES	YES	YES	YES	YES	YES
County Effect	YES	YES	YES	YES	YES	YES
*N*	2832	2832	2832	2832	2832	2832
*F*	103.5556	198.7717	59.8273	175.1312	53.1909	18.3424
*R^2^_adj*	0.3350	0.4276	0.4131	0.5695	0.1783	0.1475

Notes: *T*-statistics are in parentheses. ***, **, and * indicate significance at the 1%, 5%, and 10% levels, respectively.

## Data Availability

Not applicable.
